# PERK regulated miR-424(322)-503 cluster fine-tunes activation of IRE1 and ATF6 during Unfolded Protein Response

**DOI:** 10.1038/srep18304

**Published:** 2015-12-17

**Authors:** Ananya Gupta, Muhammad Mosaraf Hossain, Danielle E. Read, Claudio Hetz, Afshin Samali, Sanjeev Gupta

**Affiliations:** 1 Discipline of Pathology, School of medicine, Clinical Science Institute, National University of Ireland Galway, Ireland; 2 Apoptosis Research Centre, School of Natural Sciences, National University of Ireland Galway, Ireland; 3 Lambe Institute for Translational Research, National University of Ireland Galway, Ireland; 4 Biomedical Neuroscience Institute, Faculty of Medicine, University of Chile, Santiago, Chile; 5 Program of Cellular and Molecular Biology, Center for Molecular Studies of the Cell, Institute of Biomedical Sciences, University of Chile, Santiago, Chile; 6 FONDAP Center for Geroscience, Brain Health and Metabolism, Santiago, Chile

## Abstract

The endoplasmic reticulum (ER) responds to changes in intracellular homeostasis through activation of the unfolded protein response (UPR). UPR can facilitate the restoration of cellular homeostasis, via the concerted activation of three ER stress sensors, namely IRE1, PERK and ATF6. Global approaches in several cellular contexts have revealed that UPR regulates the expression of many miRNAs that play an important role in the regulation of life and death decisions during UPR. Here we show that expression of miR-424(322)-503 cluster is downregulated during UPR. IRE1 inhibitor (4 μ8C) and deficiency of XBP1 had no effect on downregulation of miR-424(322)-503 during UPR. Treatment of cells with CCT030312, a selective activator of EIF2AK3/PERK signalling, leads to the downregulation of miR-424(322)-503 expression. The repression of miR-424(322)-503 cluster during conditions of ER stress is compromised in PERK-deficient MEFs. miR-424 regulates the expression of ATF6 via a miR-424 binding site in its 3′ UTR and attenuates the ATF6 transcriptional activity during UPR. Further miR-424 had no effect on IRE1-XBP1 axis but enhanced the regulated IRE1-dependent decay (RIDD). Our results suggest that miR-424 constitutes an obligatory fine-tuning mechanism where PERK-mediated downregulation of miR-424(322)-503 cluster regulates optimal activation of IRE1 and ATF6 during conditions of ER stress.

The endoplasmic reticulum (ER) is a multifunctional signaling organelle that controls a wide range of cellular processes. The major physiological functions of the ER include folding of membrane and secreted proteins, synthesis of lipids and sterols, and storage of free calcium^1^,^2^. Cellular stresses that impair proper folding of proteins can lead to an imbalance between the load of resident and transit proteins in the ER and the organelle’s ability to process that load. In mammals, three ER transmembrane proteins IRE1, ATF6, and PERK, respond to the accumulation of unfolded proteins in the ER lumen^1^. These sensors are bound to the ER luminal chaperone BiP (GRP78) in unstressed cells. During conditions of ER stress BiP dissociates from these sensors leading to their activation^3^. When ER stress occurs, IRE1 oligomerizes and becomes autophosphorylated, which activates it to function as an unconventional endoribonuclease^4^. Activated IRE1 excises a 26-nucleotide intron from the X-box binding protein-1 (XBP1) mRNA, which is then ligated by RtcB ligase in mammals^5^. This unconventional splicing event results in frameshift of reading codons in the XBP1 mRNA and leads to the translation of a more stable and active transcription factor, termed spliced XBP1^3^,^4^ (XBP1s). Activated PERK phosphorylates eukaryotic initiation factor 2 alpha (eIF2α), which inhibits translation to decrease the ER load. However, activating transcription factor 4 (ATF4) is preferentially translated upon the phosphorylation of eIF2α. ATF4 regulates the genes involved in redox homeostasis and amino acid metabolism^1^. During conditions of ER stress, ATF6 translocates from the ER to the Golgi, where it is processed by the site-1 and site-2 proteases (S1P and S2P), thereby releasing the N-terminal transcriptional regulatory domain into the cytoplasm^3^. The 50 kDa transcriptional domain of ATF6 translocates to the nucleus where it regulates the expression of genes with ER stress response elements (ERSE) in their promoters^6^. Thus, activation of IRE1, ATF6, and PERK initiates an ER-to-nucleus intracellular signaling cascade collectively termed unfolded protein response (UPR). The most salient feature of the UPR is to increase the transactivation function of a gamut of bZIP transcription factors, such as ATF6, ATF4 and XBP1^1^. Once activated, these transcription factors coordinate transcriptional induction of ER chaperones and genes involved in ER-associated degradation (ERAD) to enhance the protein folding capacity of the cell and to decrease the unfolded protein load of the ER, respectively.

A class of small RNAs, known as microRNAs (miRNAs), have been shown to be critically involved in many cellular processes including the control of cell survival and cell death^7^. miRNAs are generated from RNA transcripts that are exported into the cytoplasm where the precursor-miRNA molecules undergo Dicer-mediated processing thus generating mature miRNA^7^. The mature miRNA assembles into the ribonucleoprotein silencing complexes (RISCs) and guide the silencing complex to specific mRNA molecules^8^. The main function of miRNAs is to direct posttranscriptional regulation of gene expression, typically by binding to the 3′ UTR of cognate mRNAs and inhibiting their translation and/or stability^9^. Several studies have shown alterations in miRNA-expression profiles during various types of cellular stresses^10^,^11^. Argonaute family member EIF2C2 (Ago2), a vital component of RISCs is distributed diffusely in the cytoplasm and redistributes from cytoplasm to stress granules and processing (P)-bodies upon exposure to stress conditions^12^. Stress-induced enrichment of Ago2 from cytoplasm to P-bodies is dependent on mature miRNAs suggesting a link between miRNAs and cellular stress^12^. Global approaches in different cellular contexts have revealed that ER stress modifies the expression of many miRNAs^13^. Loss of miRNA biogenesis has been shown to provide resistance to ER stress-induced apoptosis^14^,^15^. The complex relationship between miRNAs and ER stress pathways is only beginning to be experimentally dissected. The model emerging from these studies is that miRNAs fine tune the ER stress machinery and modulate cellular adaptation to stress.

miR-424 (miR-322 in rodents) and miR-503 are co-transcribed as a polycistronic primary transcript (pri-miRNA) and thus comprise the miR-424(322)-503 gene cluster^16^. miRNAs belonging to miR-424(322)-503 cluster have AGCAGC as the seed sequence and are part of the miR-16 family^16^. The role of miR-424(322)-503 cluster has been investigated in several types of physiological and pathological conditions, which revealed the diverse function of miR-424(322) in different situations^16^,^17^. The miR-424(322)-503 cluster promotes differentiation and induces G1 arrest by targeting an overlapping set of cell cycle regulators^18^,^19^. Expression of miR-503 in human endothelial cells (EC) results in cell cycle delay in G1, reduced cell proliferative and migratory capacity, and impaired EC networking capacities, suggesting an anti-angiogenic role for this miRNA cluster^16^,^17^. The expression of miR-424(322)-503 cluster is regulated by TGF-β in mammary epithelial cells where it regulates remodelling of the epithelium in the involuting mammary gland^20^. Deregulation of miR-424(322)-503 cluster is commonly observed in several tumour types however there are conflicting results regarding its prognostic role^16^,^21^. The miRNA belonging to miR-424(322)-503 cluster have distinct functions in different cell types but the underlying molecular mechanisms are still unclear.

In this study we focused on the role of miR-424(322)-503 cluster in ER stress responses. Our data indicate that expression of miRNAs belonging to miR-424(322)-503 is reduced during conditions of ER stress in a variety of cell types. We show that the miR-424(322)-503 cluster is repressed in a PERK-dependent manner. We provide evidence that miR-424 regulates the ER stress-mediated activation of transcriptional activity of ATF6 and RIDD activity of IRE1. Finally, we show that miR-424 regulates the expression of ATF6 via a miR-424 binding site in the 3′ UTR of ATF6. Taken together, our study revealed a novel function of PERK in the regulation of ATF6 activity and RIDD activity of IRE1.

## Results

### Downregulation of miR-424(322)-503 cluster during conditions of UPR

**Figure 1:**
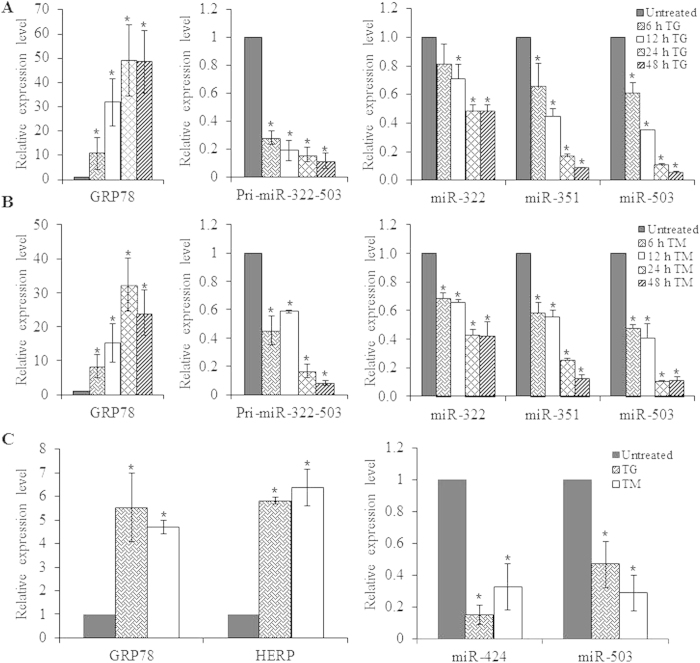
Downregulation of miR-424(322)-503 cluster during conditions of ER stress. (**A,B**) H9c2 cardiomyocytes were either untreated or treated with (1.0 μM) TG and (1.0 μg/ml) TM for 6, 12, 24 and 48 hours. The change in expression levels of ER stress marker GRP78, and primary transcript of miR-424(322)-503 cluster were quantified by qRT-PCR, normalizing against GAPDH. The expression levels of miRNAs belonging to miR-424(322)-503 cluster (miR-322, miR-351 and miR-503) were quantified by qRT-PCR, normalizing against snoRNA. The expression levels relative to the untreated control are shown. Error bars represent mean ± S.D. from three independent experiments performed in triplicate. (**C**) HEK 293T cells were either untreated or treated with (1.0 μM) TG and (1.0 μg/ml) TM for 16 hours. The change in expression levels of GRP78 and HERP (ER stress markers) were quantified by qRT-PCR, normalizing against GAPDH. The expression levels of miRNAs belonging to miR-424(322)-503 cluster (miR-322 and miR-503) were quantified by qRT-PCR, normalizing against snoRNA. The expression levels relative to the untreated control are shown. Error bars represent mean ± S.D. from three independent experiments performed in triplicate. *P < 0.05, two-tailed unpaired t-test as compared to untreated control.

**Figure 2:**
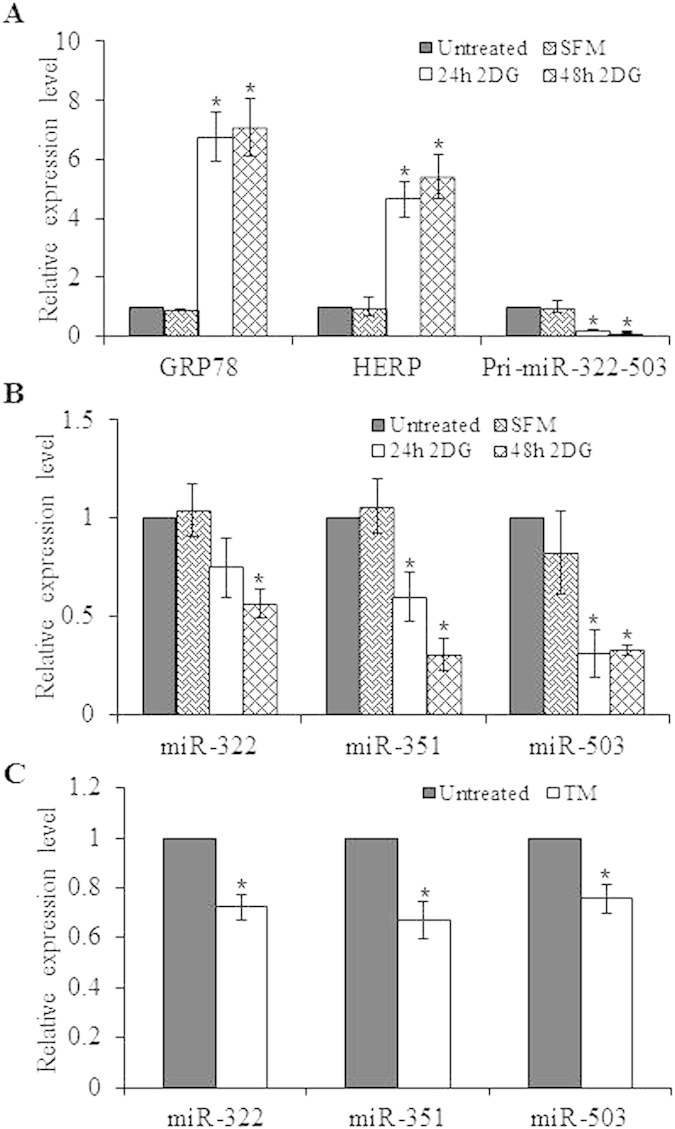
Downregulation of miR-424(322)-503 cluster in liver during conditions of systemic ER stress. (**A,B**) H9c2 cells were treated with 2-deoxyglucose (1 mM) along with serum and glucose deprivation for 24 and 48 hours. (**A**) The change in expression levels of GRP78, HERP and primary transcript of miR-424(322)-503 cluster were quantified by qRT-PCR, normalizing against GAPDH. (**B**) The expression levels of miRNAs miR-322, miR-351 and miR-503 were quantified by qRT-PCR, normalizing against snoRNA. Error bars represent mean ± S.D. from three independent experiments performed in triplicate. *P < 0.05, two-tailed unpaired t-test as compared to untreated control. SFM, serum free medium; 2DG, 2-deoxyglucose. (**C**) The total RNA from liver of untreated or tunicamycin (TM) treated mice was used to determine the expression levels of miR-322, miR-351 and miR-503 by qRT-PCR, normalizing against snoRNA. *P < 0.05, two-tailed unpaired t-test as compared to untreated control.

In preliminary experiments, the relative abundance of miRNAs comprising the Sanger miRBase database (Release 11.0) were analyzed by microarray (LC sciences, Houston, TX, USA) using RNA from H9c2 cells during conditions of ER stress^13^. Microarray analysis revealed robust downregulation of all the miRNAs belonging to the miR-424(322)-503 cluster in H9c2 cells treated with thapsigargin (TG) or tunicamycin (TM). Thapsigargin, (an inhibitor of the sacroplasmic/endoplasmic reticulum Ca^2+^-ATPase (SERCA) pump) and tunicamycin, (an inhibitor of N-linked glycosylation) both lead to accumulation of misfolded proteins in the ER and initiate UPR^22^. miR-424(322)-503 cluster has three miRNAs (miR-322, miR-351 and miR-503) in rodents while it has two miRNAs in (miR-424 and miR-503) humans. Since the three miRNAs (miR-322, miR-351 and miR-503) are derived from the primary transcript of the miR-424(322)-503 gene, we reasoned that miR-424(322)-503 gene is transcriptionally regulated during ER stress. We evaluated the expression of miR-424(322)-503 primary transcript as well as mature miRNAs (miR-322, miR-351 and miR-503) belonging to the miR-424(322)-503 cluster. Treatment of H9c2 cells with the TG (Fig. 1A) or TM (Fig. 1B) induced mRNA levels of GRP78, a bonafide UPR-target gene. We observed that levels of miR-424(322)-503 primary transcript as well as mature miRNAs (miR-322, miR-351 and miR-503) were repressed in both TG and TM treated H9c2 cells (Fig. 1A,B). In order to confirm that regulation of the miR-424(322)-503 cluster during ER stress was not restricted to H9c2 cells, we examined levels of miR-424 and miR-503 during conditions of ER stress in 293T cells. Treatment of 293T cells with TG or TM induced mRNA levels of GRP78 and HERP, bonafide UPR-target genes (Fig. 1C) whilst expression levels of miR-424 and miR-503 were repressed in both TG and TM treated 293T cells (Fig. 1C). Glucose deprivation is one of the crucial physiologic conditions leading to UPR activation, which is associated with several human diseases including tissue ischemia and cancer^23^. H9c2 cells were subjected to a combination of serum and glucose deprivation as described in methods section. We observed that glucose deprivation induced the expression of GRP78 and HERP (UPR target genes), thereby confirming the induction of UPR (Fig. 2A) upon glucose deprivation. We found that conditions of glucose deprivation reduced the levels of primary transcript as well as mature miRNAs of miR-424(322)-503 cluster in H9c2 cells (Fig. 2A,B). Next we determined the expression of miR-322, miR-351 and miR-503 *in vivo* in the liver of mice undergoing systemic ER stress. To induce systemic ER stress *in vivo*, mice were administered with tunicamycin intraperitoneally, as described in method section. We observed that levels miR-322, miR-351 and miR-503 were repressed in the liver of animals injected with tunicamycin as compared to untreated animals (Fig. 2C). These results show the downregulation of miR-424(322)-503 cluster during the conditions of ER stress.

### Repression of miR-424(322)-503 during ER stress is PERK-dependent

**Figure 3:**
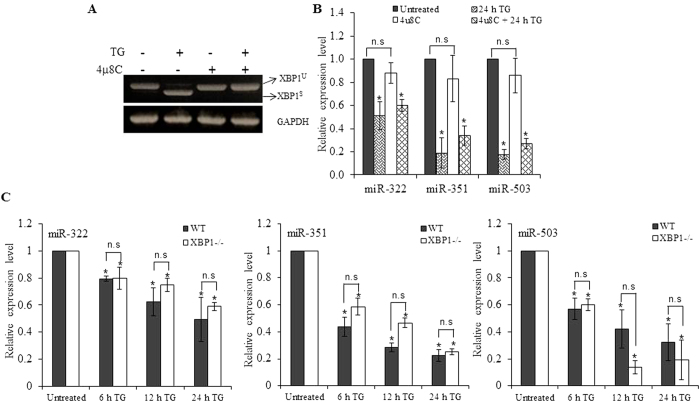
Downregulation of miR-424(322)-503 cluster during ER stress is independent of IRE1-XBP1 axis. (**A,B**) H9c2 cells were either untreated or treated with (1.0 μM) TG, (50 mM) 4 μ8C alone, TG and 4 μ8C for 24 hours. Cells were harvested and XBP1 gene expression was analysed by PCR followed by gel electrophoresis. The expression levels of miR-322, miR-351 and miR-503 were quantified by qRT-PCR, normalizing against snoRNA. The expression levels relative to the untreated control are shown. Error bars represent mean ± S.D. from three independent experiments performed in triplicate. (**C**) Mouse Embryonic Fibroblasts (MEFs) cells from wild-type or XBP1 knockout mouse were either untreated or treated with TG (1.0 μM) for 6, 12 and 24 hours and the expression levels of miR-322, miR-351 and miR-503 were analysed by qRT-PCR. The expression of miRNA was relative to snoRNA. *P < 0.05, two-tailed unpaired t-test as compared to untreated control.

Next we evaluated the roles of IRE1 and PERK in downregulation of miR-424(322)-503 cluster during UPR. We treated H9c2 cells with TG in absence and presence of IRE1 inhibitor, 4 μ8C^24^. First we evaluated the effect of 4 μ8C on induction of spliced XBP1. As shown in Fig. 3A, 4 μ8C efficiently attenuated the production of spliced XBP1. This confirms that 4 μ8C is blocking the RNase activity of IRE1. However under similar conditions 4 μ8C had no effect on the downregulation of miR-322, miR-351 and miR-503 (Fig. 3B). Next we treated wild-type (WT) and XBP1 knockout mouse embryonic fibroblasts (MEFs) with TG. We observed that repression of miR-322, miR-351 and miR-503 was comparable in WT and XBP1 knockout MEFs (Fig. 3C). These results suggest that downregulation of miR-322, miR-351 and miR-503 during UPR is independent of IRE1-XBP1 axis.

**Figure 4:**
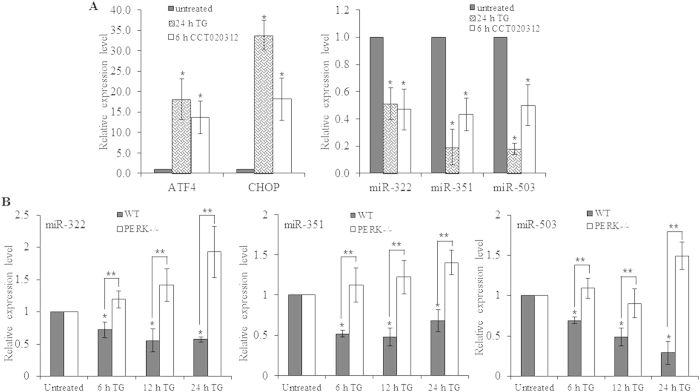
PERK-dependent regulation of miR-424(322)-503 cluster during conditions of ER stress. (**A**) H9c2 cells were either untreated or treated with TG (1.0 μM) for 24 hours or CCT030312 (10 μM) for 6 hours. The change in expression levels of ATF4 and CHOP were quantified by qRT-PCR, normalizing against GAPDH. The expression levels of miR-322, miR-351 and miR-503 were quantified by qRT-PCR, normalizing against snoRNA. The expression levels relative to the untreated control are shown. Error bars represent mean ± S.D. from three independent experiments performed in triplicate. (**B**) MEFs cells from wild-type or PERK knockout mouse were either untreated or treated with TG (25 nM) for 6, 12 and 24 hours and the expression levels of miR-322, miR-351 and miR-503 were analysed by qRT-PCR. The expression of miRNA was relative to snoRNA. The error bar represents mean ± S.D. *P < 0.05, two-tailed unpaired t-test as compared to untreated control; **P < 0.05, two-tailed unpaired t-test comparing the induction in WT and PERK −/− MEFs.

To determine the role of PERK we used CCT020312, a selective activator of EIF2AK3/PERK signalling output^25^. We treated H9c2 cells with CCT020312 and TG was used as control. First we evaluated the effect of CCT020312 on induction of ATF4 and CHOP (downstream genes of EIF2AK3/PERK). Consistent with its reported activity, CCT020312-treatment resulted in induction of ATF4 and CHOP (Fig. 4A). Further induction of ATF4 and CHOP by CCT020312 was comparable to that of TG. After confirming the specific activation of EIF2AK3/PERK by CCT020312 we evaluated its effect on expression of miR-322, miR-351 and miR-503. We observed a significant repression of miR-322, miR-351 and miR-503 by CCT020312 (Fig. 4A). Next we treated wild-type (WT) and PERK knockout mouse embryonic fibroblasts (MEFs) with TG. We observed that repression of miR-322, miR-351 and miR-503 was abrogated in PERK knockout MEFs (Fig. 4B). These results suggest that downregulation of miR-322, miR-351 and miR-503 during UPR is dependent on PERK signalling.

### miR-424 modulates optimal activation of ATF6 and IRE1 during UPR

**Figure 5:**
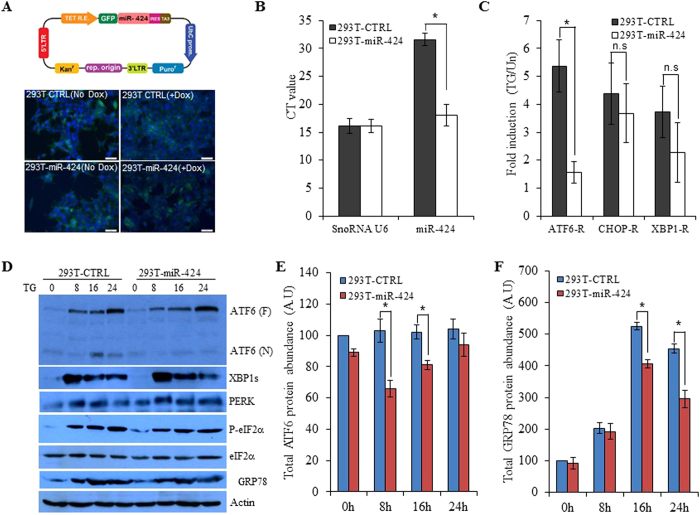
Effect of miR-424 on UPR in HEK 293T cells. (**A**) Upper panel, shows a schematic representation of lentiviral vector used to generate miR-424 expressing clones. Lower panel, expression of GFP was monitored in 293T-control and 293T-miR-424 cells after treatment with (1.0 μg/ml) of doxycycline for 24 hours. (**B**) 293T-control and 293T-miR-424 cells were treated with (1.0 μg/ml) of doxycycline for 24 h and expression levels of miR-424 was quantified by qRT-PCR, normalizing against snoRNA. Error bars represent mean ± S.D. from two independent experiments performed in triplicate. *P < 0.05, two-tailed unpaired t-test comparing the expression in 293T-control and 293T-miR-424 cells. (**C**) 293T-control and 293T-miR-424 were transfected with the indicated UPR pathway reporter gene (ATF6, CHOP, XBP1) and 24 hours post transfection cells were treated with TG (1.0 μM). Normalized Firefly luciferase activities (Firefly/Renilla) relative to untreated control are shown. *P < 0.05, two-tailed unpaired t-test comparing the increase in luciferase activity in 293T-control and 293T-miR-424 cells. (**D**) 293T-control and 293T-miR-424 cells were either untreated or treated with (1.0 μM) TG for indicated time points and immunoblotting of total protein was performed using antibodies against ATF6, spliced XBP1, PERK, GRP78, phospho-eIF2α, total eIF2α and β-actin. (**E**) Autorads obtained after probing the membrane with GRP78 antibody were analysed using Image J. Relative intensity of band representing GRP78 in 293T-miR-424 cells relative to untreated 293T-CTRL cells after normalizing aganist actin is shown. *P < 0.05, two-tailed unpaired t-test. (**F**) Autorads obtained after probing the membrane with ATF6 antibody were analysed using Image J. Relative intensity of bands representing total ATF6 [ATF6 (F) + ATF6 (N)] in 293T-miR-424 cells relative to untreated 293T-CTRL cells after normalizing aganist actin is shown. *P < 0.05, two-tailed unpaired t-test.

The 5′-end of miRNAs is an important determinant of miRNA function^26^. The miR-424(322) and miR-503 are evolutionarily conserved miRNAs of the miR-424(322)-503 cluster and possess the same seed sequence (AGCAGC)^16^. To evaluate the effect of miR-424 on UPR we generated the control (293T-CTRL) and miR-424 (293T-miR-424) overexpressing subclones of 293T cells. For this purpose 293T cells were transduced with tetracycline-inducible lentivirus engineered to produce GFP and miR-424 upon addition of tetracycline (Fig. 5A) and co-expression of the tetracycline regulatory protein, TA3. However we observed some transcriptional leakage even in the absence of doxycycline inducer in 293T-CTRL and 293T-miR-424, as determined by the expression of GFP in the absence of doxycycline (Fig. 5A). Twenty-four hours after induction with doxycycline 293T-miR-424 cells exhibited significant expression of miR-424 as compared to 293T-CTRL cells (Fig. 5B). Therefore 293T-CTRL and 293T-miR-424 clones supplemented the doxycycline (1 μg/ml) were used in subsequent experiments. Next, we tested whether miR-424 modulated the activation of three branches of the UPR. For this purpose we used synthetic luciferase reporter constructs having ATF6- or XBP1-binding sites and CHOP-promoter reporter. The induction of the XBP1-binding site and CHOP promoter reporter in response to thapsigargin was not affected by miR-424 expression (Fig. 5C). In contrast, the response of the ATF6-binding site reporter to thapsigargin was significantly decreased in 293T-miR-424 cells (Fig. 5C). Next we examined levels of UPR proteins to determine if differences existed between 293T-CTRL and 293T-miR-424 cells. Levels of ATF6, PERK, phospho-eIF2α (P-eIF2α), total- eIF2α (T-eIF2α), GRP78 and XBP1s were examined by Western blotting. No difference in UPR protein expression between 293T-CTRL and 293T-miR-424 cells was observed, with the notable exception of processed N-terminal fragment of ATF6 and GRP78 whose levels were decreased in 293T-miR-424 cells (Fig. 5D–F). Since ATF6 exists as a full length protein (ATF6-F) in the ER membranes and processed (ATF6-N) isoform in the cytoplasam and nucleus^3^. Therefore to determine the effect of miR-424 on expression of ATF6 we quantitated the expression of ATF6 (F) and ATF6 (N) in the control and miR-424 expressing cells using image J. We found that there was reduction in the total ATF6 protein in miR-424 expressing cells as compared to the control cells (Fig. 5E). These results suggest that miR-424 did not affect the PERK or IRE1-XBP1 pathway but suppressed ATF6 activity in response to ER stress.

**Figure 6:**
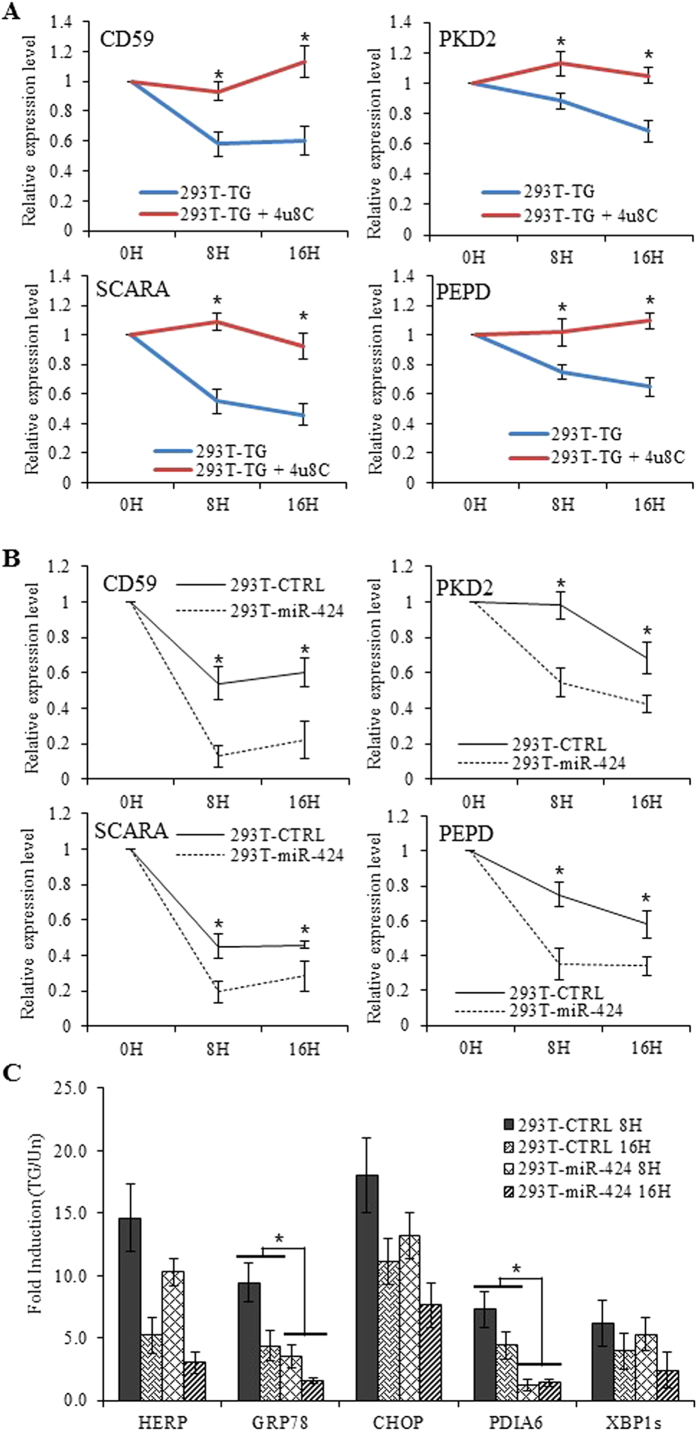
Effect of miR-424 on RIDD activity of IRE1. (**A**) 293T cells were either untreated or treated with (1.0 μM) TG in absence or presence of (50 mM) 4 μ8C for indicated time points. The change in expression levels of CD59, SCARA, PEPD and PKD2 were quantified by qRT-PCR, normalizing against GAPDH. The expression levels relative to the untreated control are shown. Error bars represent mean ± S.D. from two independent experiments performed in triplicate. *P < 0.05, two-tailed unpaired t-test. (**B**) 293T-control and 293T-miR-424 cells were either untreated or treated with (1.0 μM) TG for indicated time points. The change in expression levels of CD59, SCARA, PEPD and PKD2 (RIDD target genes) were quantified by qRT-PCR, normalizing against GAPDH. The expression levels relative to the untreated control are shown. Error bars represent mean ± S.D. from three independent experiments performed in triplicate. *P < 0.05, two-tailed unpaired t-test comparing the expression in 293T-control and 293T-miR-424 cells. (**C**) 293T-control and 293T-miR-424 cells were either untreated or treated with (1.0 μM) TG for indicated time points. The change in expression levels of GRP78, HERP, CHOP, PDIA6 and spliced XBP1 were quantified by qRT-PCR, normalizing against GAPDH. The expression levels relative to the untreated control are shown. Error bars represent mean ± S.D. from three independent experiments performed in triplicate. *P < 0.05, two-tailed unpaired t-test comparing the mRNA expression in 293T-control and 293T-miR-424 cells.

In addition to splicing of XBP1, IRE1 has been reported to cleave a subset of mRNAs that are associated with the ER membrane^27^. In these cases, unlike XBP1, IRE1-mediated cleavage is followed by degradation, a process that has been termed regulated IRE1-dependent decay^27^ (RIDD). In mammalian cells, RIDD targets are enriched for mRNAs containing a cleavage site with a consensus sequence (CTGCAG) and a predicted secondary structure similar to the conserved IRE1 recognition stem-loop of the XBP1 mRNA^28^. To evaluate the effect of miR-424 on the RIDD activity of IRE1 we determined the expression of CD59^29^, SCARA^27^, PKD2^28^ and PEPD^28^ (bonafide substrates of RIDD activity). First we confirmed that decay of CD59, SCARA, PKD2 and PEPD during UPR is dependent on RNase activity of IRE1 (Fig. 6A). We observed a time-dependent decay in mRNA levels of CD59, SCARA, PKD2 and PEPD upon TG treatment (Fig. 6B). Interestingly decay in mRNA levels of CD59, SCARA, PKD2 and PEPD in 293T-miR-424 cells was significantly enhanced as compared to 293T-CTRL cells (Fig. 6A). Analysis of gene expression profiles by quantitative RT-PCR revealed that induction of GRP78 and PDIA6 was significantly attenuated in 293T-miR-424 cells as compared to 293T-CTRL cells (Fig. 6C). Taken together, these observations suggest that miR-424 specifically regulates ER stress signaling through the modulation of transcriptional activity of ATF6 and RIDD activity of IRE1 during the UPR.

### ATF6 is a target for miR-424

**Figure 7:**
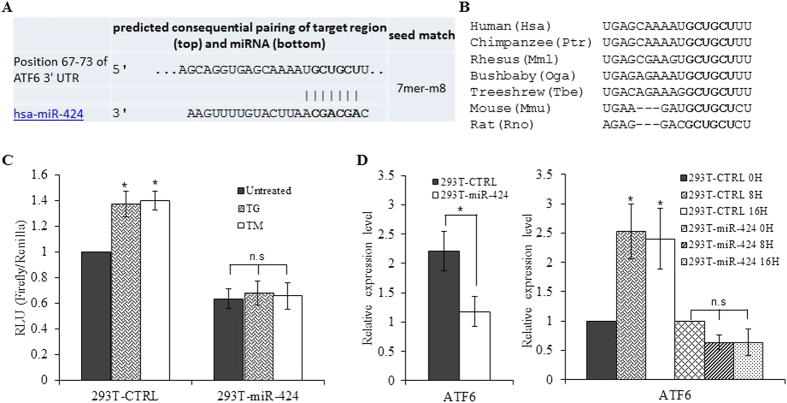
Identification of a conserved miR-424-binding site in the 3′ UTR of human ATF6. (**A**) The panel shows miR-424 binding site in the 3′ UTR of ATF6 (TargetScan). (**B**) Alignment of human ATF6 3′UTR with several mammalian species highlights the evolutionary conservation of the miR-424 binding site. (**C**) 293T-CTRL and 293T-miR-424 cells were transfected with ATF6 3′UTR reporter plasmid containing binding sites for miR-424. 24 h after transfection cells were left either untreated or treated with (1.0 μM) TG and (1.0 μg/ml) TM for 16 h. Luciferase activity was measured 48 h after transfection using the Dual-Glo assay system and normalized luciferase activity (Firefly/Renilla) is shown. Error bars represent mean ± S.D. from three independent experiments performed in duplicate. *P < 0.05, two-tailed unpaired t-test comparing the increase in luciferase activity in 293T-control and 293T-miR-424 cells. (**D**) 293T-control and 293T-miR-424 cells were either untreated or treated with (1.0 μM) TG for indicated time points. The change in expression levels of ATF6 was quantified by qRT-PCR, normalizing against RPLP0. Error bars represent mean ± S.D. from three independent experiments performed in triplicate. *P < 0.05, two-tailed unpaired t-test comparing the mRNA expression in 293T-control and 293T-miR-424 cells.

The biological functions of miRNAs are mediated by their ability to abrogate the expression of target mRNAs. miRNAs function by binding to specific regions in the targeted mRNA, which are complementary to the seed sequence of the mature miRNA, mainly in the 3′UTR^26^. Computational prediction of targets based on seed sequence conservation identified a conserved target site for miR-424 in the 3′ UTR of ATF6 (Fig. 7A,B). In order to investigate the targeting of ATF6 by miR-424 we cloned a portion of the ATF6 3′ UTR containing the predicted wild-type binding site downstream of a luciferase reporter gene. We observed that basal luciferase activity of ATF6 3′UTR construct was significantly reduced in 293T-miR-424 cells as compared to 293T-CTRL cells (Fig. 7C). Moreover treatment with ER stress-inducing agents- TG and TM caused an increase in the luciferase activity of ATF6 3′UTR construct in 293T-CTRL cells but not in 293T-miR-424 cells (Fig. 7C). A recent study using quantitative mass spectrometry and microarray analysis to determine the global effect of miRNAs on the abundance of proteins, reported that for more highly repressed targets, mRNA destabilization usually comprises the major component of repression^30^. Next we examined mRNA levels of ATF6 in 293T-CTRL and 293T-miR-424 cells. We found that basal as well as UPR-induced expression of ATF6 was significantly attenuated in 293T-miR-424 cells as compared to 293T-CTRL cells (Fig. 7D). These results suggest that miR-424 regulates the induction of ATF6 during UPR by targeting the miR-424 binding site in the 3′UTR of ATF6 mRNA.

## Discussion

**Figure 8:**
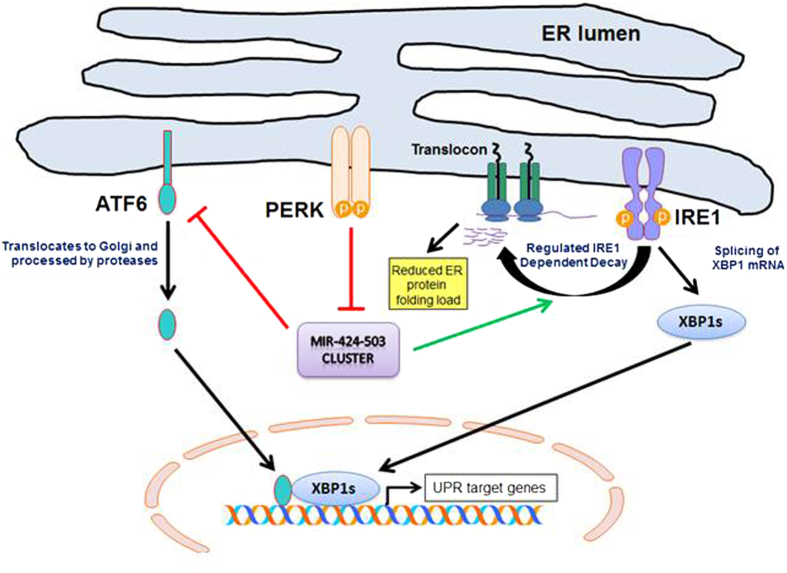
Schematic representation of the main conclusion of this manuscript. In mammals, three ER transmembrane proteins IRE1, ATF6, and PERK, respond to the accumulation of unfolded proteins in the ER lumen. Our results suggest that expression of miRNAs belonging to miR-424(322)-503 is reduced during conditions of ER stress in a PERK-dependent manner. The miR-424(322) down regulates the expression of ATF6 via a miR-424 binding site in the 3′ UTR of ATF6 and represses the ER stress-mediated increase in transcriptional activity of ATF6. The miR-424(322) regulates the RIDD activity of IRE1 but has no significant effect on induction of spliced XBP1. Taken together our results suggest that PERK-mediated downregulation of miR-424(322)-503 cluster regulates optimal activation of IRE1 and ATF6 during conditions of ER stress.

ER stress constitutes a physiological as well as pathological stress stimulus, which when overwhelming, can lead to apoptotic death of the damaged cell^31^. This study demonstrates that PERK-dependent repression of miRNAs belonging to the miR-424(322)-503 cluster play an important role in the ER stress-response through regulation of the ATF6 and IRE1 arms of UPR (Fig. 8). Several studies have revealed a role for PERK regulated miRNAs in the mediation of UPR^32^,^33^. PERK has been implicated in the induction as well as downregulation of certain miRNAs during UPR. For example, expression of miR-708 and miR-211 is induced during UPR by C/EBP homologous protein (CHOP)^33^,^34^. The expression of miR-106b-25 cluster and miR-199a-214 is repressed in a PERK-dependent manner during UPR^32^,^35^. In this work, we have identified the miR-424(322)-503 cluster as miRNAs that respond to ER stress in a PERK-dependent fashion (Fig. 4). The most likely mechanism is transcriptional repression by PERK-regulated transcription factors. Walter and colleagues have reported downregulation of miR-(424)322-503 cluster in wild-type and CHOP^−/−^ MEFs during conditions of UPR, thereby ruling out the role of CHOP^34^. Bajic’s group has identified binding sites for (RUNX1, E2F3, SP3, YY1, NFE2L2, CREB1, ATF2, USF2, ELK1, CEBPB and HOXA4) in the promoter region of miR-424-503 cluster^36^. Further time-lagged expression correlation analysis demonstrated that NFE2L1 and ATF2 were negatively correlated to miR-424 during monocytic differentiation^36^. Interestingly activity of ATF2 and NFE2L2 is regulated by PERK during UPR^3^. We propose that ATF2 and NFE2L2 might play a role in repression of miR-(424)322-503 cluster during UPR. However, further work is required to elucidate the molecular details of miR-424(322)-503 cluster downregulation during UPR. We have used a variety of cell lines in this study, for example H9c2 (rat cardiomyocytes), mouse embryonic fibroblasts, and HEK 293T (human embryonic kidney), which all possess different features and observed reduced expression of miRNAs belonging to miR-424(322)-503 cluster during UPR. Our results suggest that downregulation of the miR-424(322)-503 cluster in the response to ER stress is a general event.

This report provides evidence that miR-424(322) regulates the ER stress-mediated induction of transcriptional activity of ATF6 and RIDD activity of IRE1. Moreover, our results show that miR-424 directly regulates the ATF6 expression via a miR-424 binding site in the 3′ UTR of ATF6. These results underscore the importance of posttranscriptional modifications in regulating the optimal activation of ATF6 and IRE1 arms of UPR during ER stress. The UPR-associated miRNAs can fine tune the UPR, depending on their expression profile changes, specific targets, and tissue type that is involved^13^,^14^,^32^. Byrd and colleagues reported a miRNA connection between PERK and IRE1-XBP1, where miR-30c-2* is up-regulated during UPR in a PERK-dependent manner, and targets the 3′UTR of XBP1 mRNA^37^. Further XBP1(s) induced miR-346 has been shown to regulate the ER antigen transporter TAP1 and decrease the expression of this ER peptide transporter^38^. This inhibition of TAP1 expression inhibited MHC class I assembly in the ER, thereby reducing the ER protein load^38^.

Our results suggest that miR-424(322) expression can potentiate the RIDD of activity of IRE1. RIDD targets in mammalian cells exhibit a wide range of subcellular localization and biological functions and varied in different cell types^27^. A growing number of studies demonstrate that RIDD plays a role in drug and lipid metabolism in the liver^39^, neural regulation of vascular regeneration^40^, antigen presentation function of CD8α+ dendritic cells^41^, insulin synthesis in β-cells^42^ and ER stress-induced cell death^43^. The molecular mechanisms regarding the regulation of RIDD activity by miR-424(322) needs further investigations. One possible mechanism for miR-424(322) to regulate the RIDD activity of IRE1 could be via regulating the expression of PDIA6 (Protein disulphide isomerase family A, member 6). PDIA6 is a known target of miR-424(322) and modulates the duration of IRE1 signaling by physical interaction with IRE1^44^,^45^. There have been conflicting reports regarding the role of PDIA6 in regulating the activity of IRE1. PDIA6 has been shown to increase RNAse activity of IRE1^45^. However Argon and colleagues have shown that PDIA6 limits the duration of IRE1 activity and PDIA6-deficient cells exhibit sustained phosphorylation of IRE1 and splicing of XBP1 mRNA during ER stress^44^. In addition PDIA6 has been shown to negatively regulate RIDD activity of IRE1^46^. In agreement with the finding of Argon’s group our results show that expression level of PDIA6 is reduced in miR-424 cells, which also show enhanced RIDD activity (Fig. 6).

A final consideration is the potential link between miR-424(322)-503 cluster and cancer progression. Several members of the miR-16 family have been shown to be involved in human cancers^21^. Interestingly, miR-424(322)-503 cluster is located in a chromosomal region that has been shown to harbour deletions in 8% of luminal breast cancer^47^. Thus, one unanswered question is whether downregulation of miR-424(322)-503 due to activation of UPR in the tumour microenvironment is involved in development of breast cancer. Indeed miR-424(322)-503-KO female mice develop alveolar hyperplasia, associated with increased proliferation and reduced apoptosis^20^. Further research will be necessary to fully define the potential role of UPR mediated downregulation of miR-424(322)-503 in breast cancer progression.

## Methods

### Cell culture and treatments

MEFs, H9c2 and HEK 293T cells were maintained in Dulbecco’s modified medium (DMEM) supplemented with 10% FCS, 100 U/ml penicillin and 100 mg/ml streptomycin at 37 °C with 5% CO_2_. XBP1−/− and wild type control MEFs were a gift from Dr. Laurie Glimcher, Harvard Medical School, USA. PERK +/+ and PERK−/− MEFs were a gift from Dr. David Ron (Institute of Metabolic Science, University of Cambridge, UK). PERK +/+ and PERK−/− MEFs were maintained in DMEM medium supplemented with 10% fetal bovine serum, 2 mM glutamine, 1 mM sodium pyruvate, non-essential amino acid solution, 55 μM β-mercaptoethanol (βME), 1% penicillin/streptomycin at 37 °C with 5% CO_2_. To induce ER stress, cells were treated with tunicamycin (TM) or thapsigargin (TG) at the indicated concentrations for the indicated time. Glucose deprivation was achieved by changing the serum and glucose-containing DMEM to serum and glucose free-DMEM and (1 mM) 2-deoxyglucose for 24–48 hours. To inhibit IRE1 endoribonuclease activity, cells were treated with IRE1 inhibitor (4 μ8C) (Cat # 412512 Millipore Ireland B.V.). To specifically activate PERK cells were treated with EIF2AK3 Activator (Cat # 324879 Millipore Ireland B.V.). All reagents were purchased from Sigma–Aldrich unless otherwise stated.

### Generation of stable cell lines

We generated stable subclones of expressing miR-424 by transducing the HEK 293T cells with lentivirus made using control and miR-424 expression vector pLenti-III-Tet-mir (Applied Biological Materials Inc) and puromycin selection (3 μg/ml) for 7 days. This lentivector is designed to induce the expression of GFP and miRNA of interest upon addition of tetracycline.

### Animals and surgical procedures

The study was carried out in strict accordance with the recommendations in the Guide for the Care and Use of Laboratory Animals of the National Institutes of Health. The protocol was approved by the Committee on the Ethics of Animal Experiments of the University of Chile (CBA#0265 FMUCH). All surgery was performed ketamine/xylazine anesthesia, and all efforts were made to minimize suffering. Mice were housed under a 12:12 h light-dark cycle with access to food and water *ad libitum*.

For induction of ER stress *in vivo*, mice received a single intraperitoneal injection of tunicamycin (5 mg/kg) diluted in sterile 150 mM glucose solution as described previously^48^,^49^. Control mice received intraperitoneal injection of vehicle (5% DMSO in 150 mM glucose solution). Mice were euthanized and tissue collected 24 h after injection. Total RNA from tissues was isolated using Trizol as recommended by the supplier (Life Technologies, 15596-018).

RNA extraction, RT-PCR and real time RT-PCR—Total RNA was isolated using Trizol (Life Technologies) according to the manufacturer’s instructions. Reverse transcription (RT) was carried out with 2 μg RNA and random primerss (Promega) using ImProm-II™ Reverse Transcription System (Promega). Real-time PCR method to determine the induction of UPR target genes has been described previously^50^. Briefly, cDNA products were mixed with 2 × TaqMan master mixes and 20 × TaqMan Gene Expression Assays (Applied Biosystems) and subjected to 40 cycles of PCR in StepOnePlus instrument (Applied Biosystems). Relative expression was evaluated using the ΔΔCT method.

Measurement of miRNA Levels Using TaqMan qRT-PCR Assays—Total RNA was reverse transcribed using the TaqMan miRNA Reverse Transcription Kit and miRNA-specific stem-loop primers (Applied BioSystems) and has been described previously^22^.

### Luciferase reporter Assays

In promoter assays, 293T-CTRL and 293T-miR-424 cells were transfected with (1.0 μg) firefly luciferase vectors in combination with (100 ng) Renilla luciferase vector as internal control. 5xATF6-pGL3 (ATF6 pathway reporter) has five copies of ATF6-binding sites CTCGAGACAGGTGCTGACGTGGCGATTC cloned into pOFlucGL3 upstream of the c-fos minimal promoter (−53 to +45 of the human c-fos promoter) was obtained from Addgene (Plasmid #11976). pGADD153-luc (PERK pathway reporter) has a 804-bp GADD153 promoter sequence cloned in pGL3-basic was a gift from Professor Stephen B. Howell, University of California, USA. 4xXBP1-PGL3 (IRE1-XBP1 pathway reporter) has 4 copies of XBP-1 binding site 5′-CGCG(TGGATGACGTGTACA)_4_-3′ cloned in the -40-Luc plasmid and was a gift from Dr Laurie Glimcher, Harvard Medical School, USA. Twenty four hours post-transfection cells were treated with thapsigargin or tunicamycin for 24 h. Firefly luciferase and Renilla luciferase activities were measured 48 h after transfection using Lucetta™ Luminometer (Lonza) and then normalized for Renilla luciferase activity. In ATF6 3′UTR reporter assays, 293T-CTRL and 293T-miR-424 cells were transfected with (1.0 μg) pMirTarget vector containing the ATF6 3′UTR (Cat # SC205479 Origene Technologies) along with (100 ng) Renilla luciferase vector as internal control. At 24 h after transfection cells were treated with TG or TM for 24 h. Firefly luciferase and Renilla luciferase activities were measured using Lucetta™ Luminometer and then normalized for Firefly luciferase activity.

### Western blotting

Cells were washed once in ice-cold PBS and lysed in whole cell lysis buffer (20 mM HEPES pH 7.5, 350 mM NaCl, 0.5 mM EDTA, 1 mM MgCl2, 0.1 mM EGTA and 1% NP-40) after stipulated time of treatments and boiled at 95 °C with Laemmli’s SDS-PAGE sample buffer for 5 min. Protein concentration was determined by Bradford method. Equal amounts (20 μg/lane) of protein samples were run on an SDS polyacrylamide gel. The proteins were transferred onto nitrocellulose membrane and blocked with 5% milk in PBS-0.05%Tween. The membrane was incubated with the primary antibody for ATF6 (Abcam, Cat# ab122897), spliced XBP1 (Biolegend, Cat# 619502), PERK (Cell signaling, Cat# C33E10), GRP78 (Pierce antibodies, Cat# PA1-014A), phospho-eIF2α (Cell signaling, Cat# 9721), total eIF2α (Cell signaling, Cat# 9722) and or β-Actin (Sigma, Cat# A-5060) overnight at 4 °C. The membrane was washed 3 times with PBS-0.05% Tween and further incubated in appropriate horseradish peroxidase-conjugated secondary antibody (Pierce) for 90 min. Signals were detected using Western Lightening Plus ECL (Perkin Elmer).

### Statistical Analysis

The data is expressed as mean ± SD for three independent experiments. Differences between the treatment groups were assessed using Two-tailed paired student’s t-tests. The values with a p < 0.05 were considered statistically significant.

## Additional Information

**How to cite this article**: Gupta, A. *et al.* PERK regulated miR-424(322)-503 cluster fine-tunes activation of IRE1 and ATF6 during Unfolded Protein Response. *Sci. Rep.* **5**, 18304; doi: 10.1038/srep18304 (2015).
